# Concomitant Crohn’s Disease and Anti-Glutamic Acid Decarboxylase (GAD)-Associated Autoimmune Encephalitis: A Case Report

**DOI:** 10.7759/cureus.45344

**Published:** 2023-09-16

**Authors:** Erkam Akkoyun, Filiz Akyuz, Bilger Cavus, Tuncay Gunduz, Murat Kürtüncü

**Affiliations:** 1 Department of Neurology, Istanbul Faculty of Medicine, Istanbul University, Istanbul, TUR; 2 Department of Gastroenterology, Istanbul Faculty of Medicine, Istanbul University, Istanbul, TUR; 3 Department of Neurology, Istanbul University, Istanbul Faculty of Medicine, Istanbul, TUR

**Keywords:** cerebrospinal fluid (csf), intravenous immunoglobulins (ivig), crohn’s disease, anti-gad antibody, autoimmune encephalitis

## Abstract

Crohn's disease is an inflammatory, autoimmune disorder that predominantly affects the intestines but can also affect extraintestinal organs. Certain neurological conditions, such as autoimmune encephalitis, can develop along with this disease. In this case report, we present a case of anti-glutamic acid decarboxylase (GAD) antibody-associated autoimmune encephalitis that occurred shortly after the diagnosis of Crohn's disease and was unrelated to the treatment and nutritional deficiencies. After a significant weight loss (24 kg) and persistent diarrhea, the patient was diagnosed with Crohn's disease by colonoscopy and biopsy. Within two weeks after the diagnosis, he experienced altered consciousness and memory impairment, followed by a rapid deterioration in consciousness and respiratory distress, leading to intubation and admission to the intensive care unit. His brain MRI revealed asymmetrical diffuse cortical diffusion restrictions, hyperintense signals on fluid-attenuated inversion recovery (FLAIR) sequences, and diffuse pachymeningeal contrast enhancement involving both cingulate gyri, bilateral insular cortices, amygdalae, hippocampi, and the right precuneus. Analysis of cerebrospinal fluid (CSF) revealed a slight elevation of CSF proteins, and the patient tested positive for serum anti-GAD antibodies. The patient responded favorably to a seven-day course of intravenous methylprednisolone, five days of intravenous immunoglobulin (IVIG), and oral corticosteroids. Subsequent treatment consisted of monthly IVIG, azathioprine, and vedolizumab, resulting in no neurologic sequelae except mild amnesia. A follow-up MRI at three months showed a nearly complete disappearance of the lesions. This is the first reported case of anti-GAD-associated encephalitis occurring in the presence of Crohn's disease.

## Introduction

Crohn's disease is an idiopathic inflammatory disorder that primarily affects the digestive tract but can also exhibit extraintestinal symptoms. Neurological complications are documented in 0.2% to 35.7% of Crohn's disease patients [1], especially in patients on anti-tumor necrosis factor (TNF) therapy [2], or attributed to Wernicke syndrome caused by vitamin B1 deficiency [3]. However, the central nervous system involvement in Crohn’s disease patients without a history of immunomodulatory treatments or dietary deficits [4], autoimmune encephalitis caused by anti-N-methyl-D-aspartate-receptor (NMDAR) [2], and anti-myelin oligodendrocyte glycoprotein (MOG) antibody-associated disease [5] have been reported recently. Herein, we present a case with anti-GAD-associated autoimmune encephalitis that developed shortly after the onset of Crohn's disease. Notably, the patient responded favorably to immunomodulatory therapy.

## Case presentation

**Figure 1:**
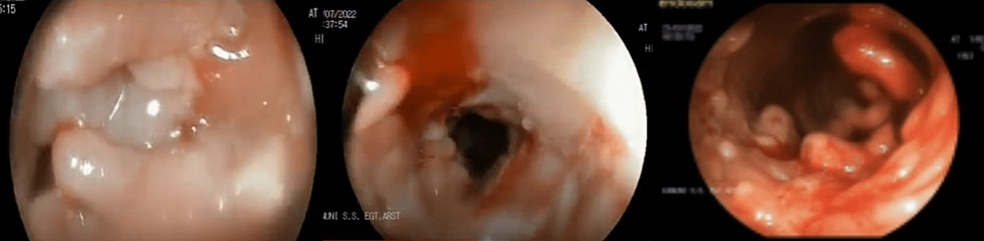
Fistula and deep ulcer detected in the anal canal, multiple pseudopolyps, and segmental ulceration in the colon.

**Figure 2:**
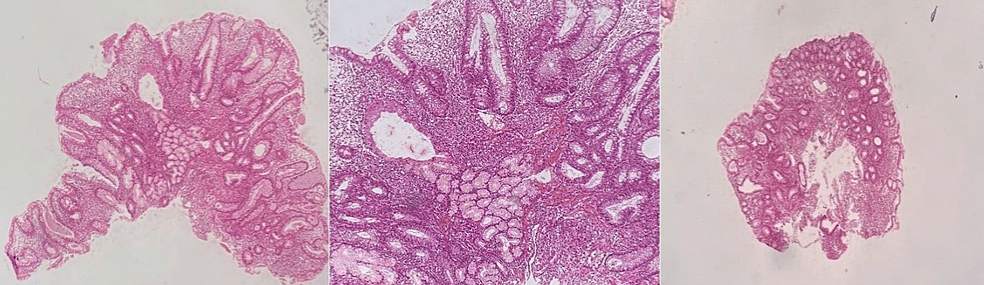
Irregular crypts, extensive lamina propria due to inflammation, and pseudopyloric metaplasia in the middle, presence of pseudopyloric metaplasia area in the lower middle, inflamed mucosa, and crypt abscess showing crypt irregularity.

Significant weight loss (24 kg) and chronic diarrhea complaints in a 23-year-old male patient necessitated further evaluation. Colonoscopy uncovered several pseudopolyps and segmental ulcers in the colon and fistulas and deep ulcers in the anal canal (Figure 1). The biopsies of the intestine revealed ulcers, inflammatory mucosa, crypt abscesses, and pseudopyloric metaplasia are shown in Figure 2.

**Figure 3:**
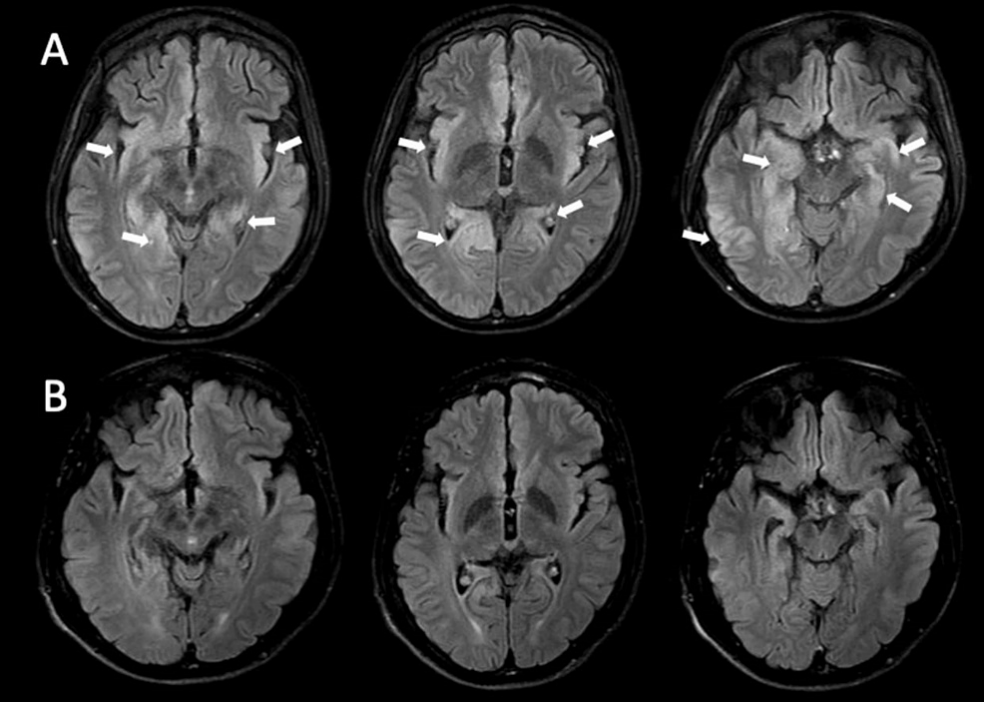
Hyperintense lesions observed in the axial FLAIR sequences, more prominent on the right side. (A) The lesions affect both cingulate gyri, bilateral insular cortices, amygdalae, hippocampi, and the right pre-cuneus. (B) After a three-month interval, axial FLAIR sequences demonstrated a substantial regression of the hyperintense lesions. FLAIR: fluid-attenuated inversion recovery

Following these investigations, the patient was diagnosed with Crohn's disease. Two weeks after the diagnosis, the patient experienced confusion, memory loss, and agitation. His symptoms rapidly progressed, leading to mechanical ventilation and an intensive care unit admission. Before intubation, the patient’s vital signs were within normal limits. His neurological evaluation indicated disorientation, no evidence of meningeal irritation, and no lateralizing symptoms; however, he had brisk deep tendon reflexes, bilateral Achilles clonus, and extensor plantar reflexes. His brain MRI revealed diffuse cortical diffusion restrictions, hyperintense fluid-attenuated inversion recovery (FLAIR) lesions, and diffuse pachymeningeal contrast enhancement involving both cingulate gyri, bilateral insular cortices, amygdalae, hippocampi, and the right precuneus (Figure 3).

All MRI findings were more prominent on the right side. Cerebrospinal fluid (CSF) biochemistry revealed glucose of 36.3 mg/dL (normal range: 40-70), total protein of 0.47 g/L (normal range: 0.15-0.45), albumin of 0.28 (normal range: 0.1-0.3), immunoglobulin G (IgG) of 0.04 g/L (normal range: 0.01-0.03), and IgG index of 0.58. CSF cytology and oligoclonal band studies were within normal limits. In addition, the CSF (BOS) meningitis and encephalitis panel yielded negative results. The patient's serum showed anti-GAD antibody levels at 49 U/mL (normal range: <5). All other anti-neuronal antibody tests, including anti-NMDA, α-amino-3-hydroxy-5-methyl-4-isoxazolepropionic acid (AMPA)-R1, AMPA-R2, contactin-associated protein-2 (CASPR2), leucine-rich, glioma-inactivated-1 (LGI1), γ-aminobutyric acid receptor (GABA-R), and dipeptidyl-peptidase-like protein-6 (DPPX), yielded negative results. When the control serum was checked again after six months, the serum anti-GAD antibody level was still high (54 U/mL).

The electroencephalogram (EEG) of the patient, who did not clinically exhibited an epileptic seizure, showed diffuse slowing in the theta and delta ranges, indicating global cerebral dysfunction. To assess for any underlying malignancy, total-body positron emission tomography (PET), thoracic and abdominal CT scans, endoscopy, and colonoscopy were performed, and all were evaluated as normal. The patient's clinical condition significantly improved following a seven-day course of intravenous methylprednisolone (IVMP) and a five-day course of intravenous immunoglobulin (IVIG), followed by oral corticosteroids. Long-term prophylactic management strategies included vedolizumab and mesalazine for Crohn’s disease and monthly IVIG and azathioprine for autoimmune encephalitis. The patient experienced no residual neurological disability, apart from mild amnesia. The subsequent brain MRI revealed an improvement concomitant with the patient's clinical recovery. The regions that had initially exhibited FLAIR hyperintensity and diffusion restriction on MRI demonstrated considerable regression after one month, ultimately approaching complete resolution during the third month.

## Discussion

The central nervous system involvement in Crohn's disease may arise from the underlying disease itself, as mentioned by Whittaker et al. In their cases, cortical hyperintensities and contrast enhancement can be observed in FLAIR MRI images. Biopsies of these lesions may reveal granulomas, and they respond well to immunosuppressive therapy [4]. In addition, in Crohn's disease patients treated with TNF-α inhibitors, both peripheral and central demyelinating lesions have been observed. Recently, Oh et al. presented a case of anti-NMDAR-associated autoimmune encephalitis in a patient on long-term infliximab therapy. While the exact mechanisms by which long-term infliximab use leads to anti-NMDAR-associated autoimmune encephalitis are not fully understood, possible explanations include the inability of TNF-α inhibitors to cross the blood-brain barrier, potentially leading to a paradoxical increase in TNF in the central nervous system. It has also been suggested that TNF-α inhibition in the intestinal mucosa may lead to a localized and then disseminated increase in autoimmune reactivity in B cells. Furthermore, the effectiveness of treatments, such as rituximab, which depletes B cells, supports these hypotheses [2]. In the context of Crohn's disease, as mentioned in the case series by Oudman et al., central nervous system involvement due to malnutrition, particularly thiamine deficiency, can occur, with severe symptoms that can be reversible with replacement therapy [3].

In the context of Crohn's disease, central nervous system involvement can manifest as various structural lesions, including cerebrovascular, demyelinating, vasculitic, and infectious, depending on the etiology. Different anatomical regions may exhibit lesions of varying sizes and characteristics in these cases [6]. In our case, there were diffuse meningeal contrast enhancement encompassing both singular gyri, bilateral insular cortices, the amygdala, the hippocampus, and the right parietal lobe, along with diffuse cortical diffusion restrictions and hyperintense lesions in FLAIR sequences. This pattern of involvement partially overlaps with the development of anti-NMDAR-associated autoimmune encephalitis following long-term infliximab therapy in the context of Crohn's disease. Different patterns of contrast enhancement can be observed in various lesions related to Crohn's disease. Recently, a study by Kuang et. al. evaluated cases of anti-GAD-associated meningoencephalitis, with four out of 25 autoimmune encephalitis patients exhibiting meningeal contrast enhancement. Meningeal contrast enhancement was attributed to the disruption of the blood-brain barrier due to local cellular inflammatory response in the leptomeninges, supported by the presence of CD3+ T cells in the leptomeninges. Their patients responded well to immunotherapy [7]. In our case, there was also diffuse contrast enhancement, and our patient responded rapidly and favorably to the immunosuppressive treatment. Therefore, this proposition was substantiated in our case.

An important point of debate is the uncertainty regarding the relationship between Crohn's disease and autoimmune encephalitis. One of these conditions may predispose the development of the other. Furthermore, it is not uncommon for individuals to develop multiple autoimmune diseases concurrently. Although there may not be a clear pathophysiological link, it is possible that both autoimmune disorders share a common etiology. Our current understanding of the potential shared pathophysiological processes between the two clinical disorders is limited, and further research is needed to elucidate the complex connection between Crohn's disease and autoimmune encephalitis.

## Conclusions

In the context of Crohn's disease, patients with cognitive dysfunction and seizures may experience encephalitis due to nutritional deficiencies and therapeutic interventions. However, beyond these factors, autoimmune encephalitis must also be considered, and anti-neuronal antibodies should be thoroughly examined. If autoimmune encephalitis is confirmed, the prompt initiation of immunomodulatory agents effective for both this condition and Crohn's disease is crucial.

## References

[1] Gondim FA, Brannagan TH 3rd, Sander HW, Chin RL, Latov N (2005). Peripheral neuropathy in patients with inflammatory bowel disease. Brain.

[2] Oh SJ, Kwon YN, Lee CK, Lee JS (2022). Anti-NMDAR encephalitis in Crohn's disease undergoing long-term infliximab treatment: a case report. Front Immunol.

[3] Oudman E, Wijnia JW, Oey MJ, van Dam M, Postma A (2021). Wernicke's encephalopathy in Crohn's disease and ulcerative colitis. Nutrition.

[4] Whittaker K, Guggenberger K, Venhoff N, Doostkam S, Schaefer HE, Fritsch B (2018). Cerebral granulomatosis as a manifestation of Crohn's disease. BMC Neurol.

[5] Philippart M, Fastré S, Rahier JF, London F (2019). First report of coexistence of MOG-antibody-positive disease and Crohn's disease. Mult Scler Relat Disord.

[6] Dolapcioglu C, Dolapcioglu H (2015). Structural brain lesions in inflammatory bowel disease. World J Gastrointest Pathophysiol.

[7] Kuang Z, Baizabal-Carvallo JF, Mofatteh M (2023). Meningoencephalitis associated with GAD65 autoimmunity. Front Immunol.

